# Combining scanning probe microscopy and x-ray spectroscopy

**DOI:** 10.1186/1556-276X-6-308

**Published:** 2011-04-07

**Authors:** Carole Fauquet, Maël Dehlinger, Franck Jandard, Sylvain Ferrero, Daniel Pailharey, Sylvia Larcheri, Roberto Graziola, Juris Purans, Aniouar Bjeoumikhov, Alexei Erko, Ivo Zizak, Brahim Dahmani, Didier Tonneau

**Affiliations:** 1 Université de la Méditerranée, CNRS-CINaM, Faculté des Sciences de Luminy, case 913, 13288 Marseille cedex 09, France; 2 AXESS TECH, 750 Chemin de Beaupré, 13760 Saint Cannat, France; 3 Dipartimento di Fisica, Universita' di Trento, Via Sommarive 14, 38123 Trento, Italy; 4 Latvian State Univ, Inst Solid State Phys, LV-1063 Riga, Latvia; 5 IFG GmbH, Rudower Chaussee 29/31, 12489 Berlin, Germany; 6 HZB-Synchrotron Bessy, Albert Einstein Strasse, 15, 12489 Berlin, Germany; 7 LovaLite, 18 Rue A.Savary, 25000 Besançon, France

## Abstract

A new versatile tool, combining Shear Force Microscopy and X-Ray Spectroscopy was designed and constructed to obtain simultaneously surface topography and chemical mapping. Using a sharp optical fiber as microscope probe, it is possible to collect locally the visible luminescence of the sample. Results of tests on ZnO and on ZnWO_4 _thin layers are in perfect agreement with that obtained with other conventional techniques. Twin images obtained by simultaneous acquisition in near field of surface topography and of local visible light emitted by the sample under X-Ray irradiation in synchrotron environment are shown. Replacing the optical fibre by an X-ray capillary, it is possible to collect local X-ray fluorescence of the sample. Preliminary results on Co-Ti sample analysis are presented.

## Introduction

Non destructive tools providing elemental and chemical analysis at high lateral resolution are needed for life and physical sciences. For example electronics or glass industries need sub-100 nm resolution tools for material processing and control (RRAM, FeRAM, smart materials, solar cells) [1]. During the last ten years, numbers of characterization tools were thus developed to obtain with the same apparatus sample imaging and chemical mapping. For example TEM (Transmission Electron Microscopy) is combined with EELS (Electron Energy Loss Spectroscopy) techniques to study oxidation states in transition metal oxides [2]. Near Field Microscopes are powerful tools for surface topography and analysis at nanometric lateral resolution. These equipments allow various in-situ spectroscopies, to probe surface local magnetic properties [3], electronic states [4] or even to identify and localize specific chemical group on very small features [5]. Combination of equipments can give further insights in sample analysis as, e.g. a combination of PEEM with STM [6]. However, those techniques are not simultaneously performed, so that authors had to mark the surface to recover the PEEM analysis localization for STM imaging at the same place.

Conventional X-Ray Absorption Spectroscopies are fine analysis techniques providing chemical and structural properties of a material, based on the spectroscopy of the emitted photons or photoelectrons. They require a high brightness X-Ray excitation source, usually a synchrotron beam, to irradiate the sample. Emergent high resolution microscopies take advantage of X-ray analysis to perform chemical mapping on samples [7]. For example, STXM (Scanning Transmission X-Ray Microscope) in transmission mode [7] and XPEEM (X-ray Photoemission Electron Microscopy) enable to obtain a sample chemical contrast and electronic structure from individual nanostructures [8,9].

Coupling X-Ray Spectroscopy and Scanning Probe Microscopy allows collecting with the microscope probe, the sample emission (electron, photons) under X-ray excitation, leading to surface topography and chemical mapping at high resolution at the same place. This concept is now widely investigated in synchrotron environment [10-13].

In this work, we present a versatile Shear Force Microscope head, which can be coupled to an X-ray beam illuminating the sample just at the level of microscope probe apex. This microscope has been fitted to a synchrotron beam line, to simultaneously perform XAFS-XEOL (X Ray Absorption Fine Structure - X Ray Excited Optical Luminescence) spectroscopy, and surface topography. A sharp optical fiber is used as microscope tip for sample topography and for local sample visible luminescence collection. Spectra exhibit the variation of the visible light intensity as a function of incident primary beam energy. As an absorption threshold, characteristic of an emitting element present in the material is crossed, the intensity of the visible light drastically increases and is followed by oscillations linked to the atomic environment and structure of this element [14]. Chemical mapping was achieved on ZnO and ZnWO_4 _- ZnO samples. μ-XRF (micro X-Ray Fluorescence) analysis was successfully carried out on Co-Ti samples, replacing the optical fibre, microscope probe, by a thin X-ray capillary and using a rotating anode (Cu Kα) as excitation source.

## Results

### Instrumentation

**Figure 1:**
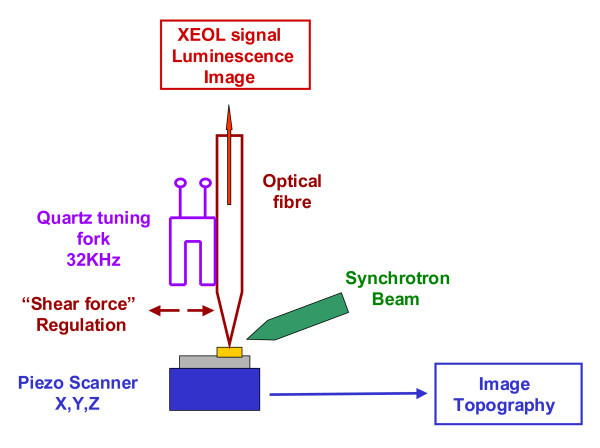
**Principle of the home-built instrument**. The instrument combines shear-force microscopy and XEOL spectroscopy.

The apparatus consists in an home-made shear-force microscope (see Figure 1) whose probe is a sharp Aluminium-coated optical fibre (aperture 50 nm) that locally collects the visible light emitted by the sample illuminated by X-Ray radiation (synchrotron environment). The instrument, working in ambient conditions or in liquid environment, allows simultaneous pixel by pixel surface topography measurement and chemical mapping [15]. The analysed sample must fit with Scanning Probe Microscopy requirements (solid sample, roughness in the micronscale range). This apparatus is evaluated by characterization of ZnO and ZnWO_4 _- ZnO thin layers, exhibiting a high luminescence yield. The luminescence spectra are compared to those obtained in far field

This apparatus also enables the XRF signal local collection of the excited sample, replacing the device tuning fork-optical fibre by a fixed X-ray cylindrical capillary (internal diameter 10 μm, length 50 mm). The sample is excited by a rotating anode (excitation at constant energy, Cu Kα at 8 keV, power 40 kV × 40 mA) while the fluorescence signal is analyzed by EDX (Energy Dispersive X-ray). The excitation beam is focused on the sample by a capillary lens (spot diameter 20 μm) provided by IFG GmbH. The XRF technique is particularly suitable for analysis of heavy elements, typically heavier than sodium.

### Nano-XAFS-XEOL

**Figure 2:**
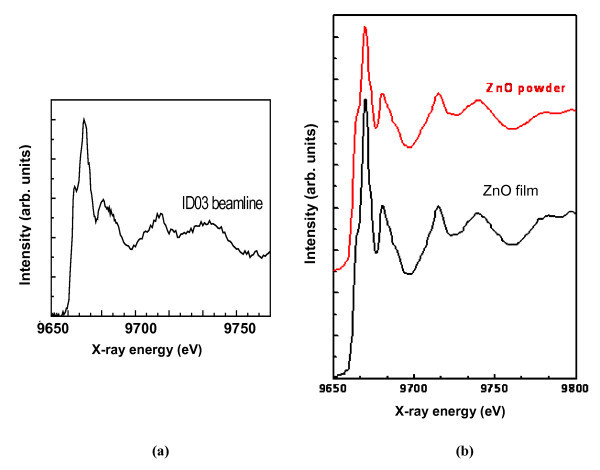
**XAFS-XEOL recorded spectra of - a: the sputtered ZnO film obtained in near-field; - b: the ZnO film (bottom) and of a reference stoichiometric ZnO powder (top) by conventional technique**. Spectra are expanded for clarity.

In Figure 2a we present the XAFS-XEOL spectrum obtained with the apparatus at ESRF ID03 line of a ZnO thin layer (~400 nm), prepared by Zn sputtering on a silicon substrate, followed by a 900°C annealing in air. The threshold, localized at 9664 eV, is characteristic of visible light emitted by Zn atoms after X-Ray absorption. This spectrum is compared with that of the same sample (Figure 2b, bottom) and with that of a commercial stoichiometric ZnO powder sample for reference (Figure 2b, top, shifted), obtained in conventional XAFS-XEOL spectroscopy, in far field, at the same beamline. Spectra shown in Figure 2b are in very good agreement in terms of both peak positions and relative magnitudes measured with respect to the average signal above threshold. This indicates that the ZnO sputtered layer is stoichiometric. The great concordance between spectra Figure 2a and Figure 2b validates the instrument concept.

**Figure 3:**
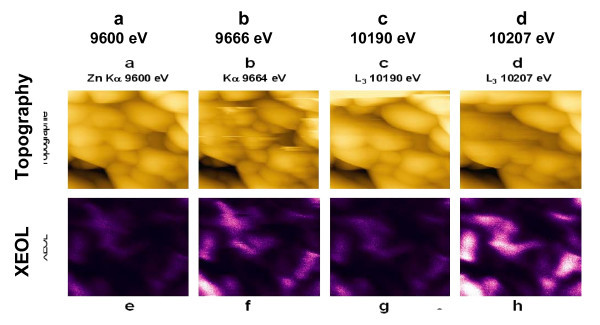
**Twin topography-luminescence images**. Top: (a-d) topography of a ZnO-ZnWO_4_ sputtered layer (2 × 2 μm^2^). Bottom: corresponding visible light emission cartography under illumination by X-ray beam from left to right below (e) and above (f) the Zn-Kα threshold (9.6 keV) and below (g) and above (h) the W-L**_3_**threshold (10.2 keV). On top of the images is indicated the X-ray primary energy.

A ZnWO_4 _- ZnO thin layer (~400 nm) was prepared by co-sputtering Zn and W onto a silicon substrate, followed by a 900°C annealing in air. In Figure 3 we show twin images corresponding to the simultaneous record of both topography and luminescence cartography of the ZnWO_4 _- ZnO sample at various incident energies. In upper Figures 3-a, b, c, d the topography is presented. Grains of 0.5 to more than 1μm are observed, as was confirmed by conventional Atomic Force Microscopy. In Figures 3-e, f, g, h we present the corresponding luminescence cartography obtained respectively, from the left to the right, before and after the Zn-K edge, as well as before and after the W-L edge. Images 3a to 3 h contain 1024 × 1024 pixels. The remarkable stability of the instrument is noticeable, since it took about 8 h for recording this whole set of images. Image 3 g, obtained at higher X-ray energy than the Zn threshold, also highlights Zn rich regions. The contrast is lower than in Figure 3f since the acquisition is performed far from the maximum emission. Black zones correspond to non emitting or to grains emitting out of the fibre acceptance angle.

**Figure 4:**
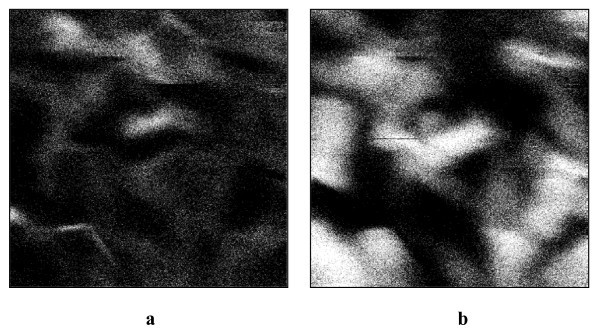
**Zn (a) and W (b) rich emitting areas of a ZnO-ZnWO_4 _thin layer deposited by magnetron sputtering**.

Post image processing can be carried out on Figure 3e to 3h to define ZnO and ZnWO_4 _rich areas. First, the pixel to pixel difference Figure 3f - Figure 3e (resp. Figure 3h - Figure 3g) gives the distribution of Zn (resp. W) luminescent sites. Then, to enhance the contrast, these two images are further converted in black and white scale. By this way we get two intermediate images, which are then used to obtain a chemical mapping of the layer: the ZnO rich emitting areas can be obtained by difference of these intermediate images (Figure 4a), since Zn is present in both materials while W can be found only in ZnWO_4 _grains. Finally, a logic operation 'AND' is applied between the intermediate images to highlight the distribution of emitting ZnWO_4 _(Figure 4b) since Zn must be present in both materials. In fact a white pixel in Figure 4b is obtained only if the same pixel appears simultaneously white on both intermediate images. This image processing leads to a two-level (black and white) image which increases significantly the contrast. Since Figure 4a shows only few features, one can conclude the emitting centres are almost pure ZnWO_4_, as confirmed by XRD and micro-Raman analysis [16]. No obvious correlation with the topography is noticeable, since the emitting zones are not specifically centered in the grains.

Collecting the XEOL signal in near field significantly increases the lateral resolution of this technique, which is now only limited by the aperture of the optical fibre. In fact, the resolution of the apparatus is limited by the tip curvature for topography (~100 nm) and by the optical aperture for the light collection (~50 nm).

### In-lab μ-XRF analysis

**Figure 5:**
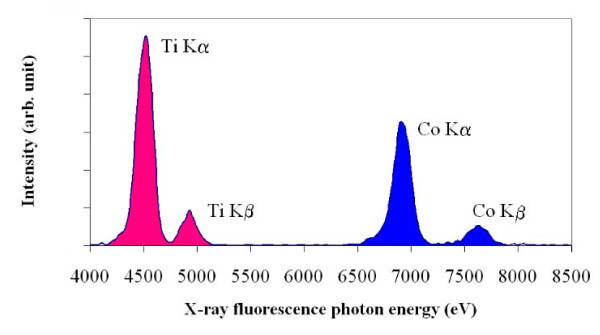
**In-lab μXRF spectra**. Typical spectrum obtained on the sample when the frontier Co-Ti is illuminated. Capillary diameter is 10 μm and the acquisition time is 100 s.

Replacing the device tuning fork-optical fibre by a fixed X-ray cylindrical capillary the XRF collection concept feasibility is demonstrated on a test sample, composed of bulk Co and Ti juxtaposed sheets. The X-ray beam simultaneously illuminates both Co and Ti samples. Figure 5 shows XRF spectra obtained using 10 μm diameter cylindrical capillary approached at a distance of 5 mm from the sample surface. We obtain the Kα and Kβ characteristic peaks of both Co and Ti, as reported in literature [17]. Since the fluorescence yield of Co is twice that of Ti at excitation energy of 8 keV, the incident spot might be slightly shifted on the titanium sheet regarding the Co-Ti separation.

With commonly marketed XRF equipment, without capillary for detection, the lateral resolution is limited by the diameter of the primary probe, in the range of 10 μm. A resolution increase can be achieved by shrinking down the detector aperture. However, increasing the resolution from 10 to 1 μm, would lead to a factor loss of 100 on the signal. To reach the original signal level, the sample-detector distance must be drastically decreased. However the steric hindrance of the EDX detector (surface of about 1 cm^2^) impedes to approach the detector at distances lower than 5 mm. Consequently, a solution to avoid primary beam shadowing is to use a low diameter capillary to collect the fluorescence signal at the vicinity of the surface. Furthermore, using for example a cylindrical capillary to collect the signal enhances significantly the signal level regarding a pinhole of the same diameter at a given sample-detector distance [18]. The gain G is given by:

Where θ_c _is the critical angle of the capillary material (in our case fused silica with θ_c _of about 5 mrad at the X-ray energy considered in this paper [19]), D is the detector-sample distance and d is the capillary diameter. G is about 3.10^3 ^(resp. 3.10^5^) for a 50 mm long and 10 μm (resp. 1 μm) diameter cylindrical capillary approached at 5 mm from the sample surface. Moreover, the use of elliptical instead of cylindrical capillary would further increase the signal level by a factor 20 [20,21]. Our experience shows that we can combine X-ray capillary optics for both excitation and detection to substantially increase the resolution of in-lab XRF technique which can be better than 1μm keeping a significant signal to noise ratio and remaining in satisfactory acquisition times [22].

### Conclusion and perspectives

We have constructed a new Shear-Force Microscopy head that is able to simultaneously record the topography and the light emitted by a sample. We have demonstrated in synchrotron environment the possibility of simultaneous XEOL mapping and surface topography with a resolution of 50 nm. The instrument is thus able to image the surface and to localize a peculiar object that can be further chemically analyzed by XEOL analysis. Thanks to the recent development of new X-Ray capillary lens, we now equip our home-made Shear Force Microscope with a tightly focused laboratory X-ray source for on-table simultaneous Luminescence-Topography measurements. The sensitivity of the technique, limited by the signal to noise ratio, will be evaluated in the future.

We have demonstrated the concept feasibility of XRF analysis at micrometer scale. In fact, replacing the optical fibre of our microscope head by a 10 μm diameter cylindrical capillary, we succeeded in local collection of sample XRF under X-ray illumination using an in-lab source. The signal level obtained in this work enables to estimate that the lateral resolution of the technique can still be improved. Consequently sub-1 μm resolution can be reached in lab, whereas, using brighter excitation sources (synchrotron), sub-100 nm resolution is expected, limited today by capillary technology. The final idea is to use an elliptic capillary as shear force probe to simultaneously obtain topography and the XRF mapping of the sample.

## Competing interests

Patent concerning the detection of XRF through capillary optics is pending (french patent n°1002392, 2010).

## Authors' contributions

FJ, SF, DP and RG were involved in instrument design and fabrication; they participated in conception and realization of light spectroscopy and microscopy experiments. DP and DT coordinated this study. BD was involved in probe conception and light collection analysis. JP, SL and DT conceived the luminescence experiments and participated in interpretation of data. JP was at the concept origin of coupling scanning probe microscopy and X-Ray spectroscopy. SL, CF and DT performed microscopy data and interpretation. MD, CF, AB and DT conceived the μ-XRF experiments and participated in acquisition and interpretation of data. AB, AE and IZ were involved in μ-XRF data discussion and interpretation. CF, MD and DT drafted the manuscript. All authors read and approved the final manuscript.
